# Effect of parecoxib on postoperative cognitive function and analgesic safety in elderly patients undergoing gastrointestinal tumor resection: A retrospective study

**DOI:** 10.17305/bb.2024.11042

**Published:** 2024-09-16

**Authors:** Yongli Li, Yan Peng

**Affiliations:** 1 Department of Anesthesiology, Hospital of Chengdu University of Traditional Chinese Medicine, Chengdu, China; 2 Department of Anesthesiology, The Third Xiangya Hospital, Central South University, Changsha, China; 3 Department of Anesthesiology, The Fourth People’s Hospital of Nanchong, Nanchong, China

**Keywords:** Parecoxib, non-steroidal anti-inflammatory drugs (NSAIDs), postoperative cognitive dysfunction (POCD), gastrointestinal tumor, analgesia, elderly

## Abstract

Neuroinflammation is associated with the development of postoperative cognitive dysfunction (POCD). Parecoxib has powerful anti-inflammatory and analgesic effects, which may reduce the occurrence of POCD. We hypothesized that parecoxib could reduce the incidence of POCD and relieve postoperative pain without increasing postoperative complications in elderly patients with gastrointestinal cancer. The study analyzed the effect of parecoxib on elderly patients undergoing elective radical resection of gastrointestinal tumors**.** Patients were divided into the non-steroidal anti-inflammatory drugs (NSAIDs) group and the non-NSAIDs group according to whether parecoxib was administered. Demographic and clinical data were collected and compared. The incidence of POCD was set as the primary outcome, and postoperative pain as the secondary outcome. Among the 440 enrolled patients, the POCD incidence rates within seven days after surgery in the NSAIDs and non-NSAIDs groups were 42.60% and 40.30%, respectively, with no statistically significant difference (*P* > 0.05). Patients in the NSAIDs group experienced significantly less pain on the first and second days after surgery compared to the non-NSAIDs group (*P* < 0.05). There were no statistically significant differences in postoperative adverse events between the two groups (*P* > 0.05). Parecoxib had no significant negative effect on early postoperative cognitive function, effectively alleviating early postoperative acute pain without increasing postoperative complications. The findings have implications for the broader use of parecoxib in postoperative pain management in elderly patients undergoing major surgery.

## Introduction

Postoperative cognitive dysfunction (POCD) represents a prevalent form of cognitive impairment, particularly observed in elderly patients over 65 years of age [1–3]. The cognitive decline in POCD is particularly pronounced in areas related to executive function, memory, judgment, logical analysis, and orientation. Long-term effects can lead to early retirement, increased mortality, and an increased healthcare burden. According to previous studies, the incidence of POCD ranges from 17% to 43% [4]. Advanced age, inflammation, and preoperative cognitive impairment have been reported as potential risk factors for POCD [2]. POCD diagnosis is assessed using a series of neuropsychological tests; however, criteria vary. Currently, the pathological mechanisms and treatments for POCD have not been fully elucidated. Hence, an effective approach may involve proactive measures aimed at averting these risk factors.

Neuroinflammation is an important mechanism underlying POCD [5]. Notably, studies have shown that increased levels of pro-inflammatory cytokines after surgery are associated with the development of POCD [6–8]. Furthermore, pro-inflammatory cytokines can breach the blood–brain barrier and enter the central nervous system. Lu et al. [9] reported that pretreatment with parecoxib sodium in conjunction with dexmedetomidine may lead to a reduction in the incidence of early POCD among elderly patients. A meta-analysis that included 35 randomized trials indicated that parecoxib may be effective in preventing POCD on postoperative day 3 [10]. Tian et al. [11] discovered that pre-emptive analgesia administered using 40 mg of parecoxib sodium is effective in reducing the occurrence of POCD among elderly patients. Therefore, the potential modulation of anti-inflammatory pathways through non-steroidal anti-inflammatory drugs (NSAIDs) like parecoxib may be brain protective and reduce the incidence of POCD [12, 13]. NSAIDs are widely used in the perioperative period and are known to have analgesic, antipyretic, and immunomodulatory effects by inhibiting cyclooxygenase and reducing prostaglandin synthesis, however, NSAIDs can adversely affect multiple organ systems, such as the digestive tract, kidneys, and heart, which limits their clinical application [14].

This study aimed to explore the effects of NSAIDs, specifically parecoxib, on POCD and their analgesic safety in elderly patients undergoing radical resection of gastrointestinal tumors. Parecoxib is a cyclooxygenase-2 inhibitor and is different from aspirin. Given the known role of inflammation in POCD and the anti-inflammatory properties of NSAIDs, there is growing interest in investigating whether parecoxib can mitigate the development of POCD in this population. Furthermore, assessing the safety of parecoxib for analgesia in these patients is essential, as it has the potential to provide valuable insights into their perioperative care and overall well-being.

## Materials and methods

### Study population

This was a retrospective study. This experiment was approved by the Ethics Committee of Xiangya Third Hospital (No: Fast | 21011). This study included elderly patients (aged ≥ 65 years) with gastrointestinal tumors who underwent selective radical surgery under intravenous–inhalation combined anesthesia in our hospital from January 2018 to May 2021. The inclusion criteria were: (1) Patients planning to undergo selective gastrointestinal resection under general anesthesia; (2) Age ≥ 65 years old; (3) Preoperative mini-mental state examination (MMSE) scores ≥ 24 points; (4) The anesthesia protocol was intravenous–inhalation combined with general anesthesia; and (5) The surgical procedure was performed laparoscopically. The exclusion criteria were: (1) A history of mental or neurological illness; (2) A history of using sedatives or antidepressants; (3) Serious sensory barriers that affect the assessment; (4) Patients who received analgesics other than parecoxib during surgery or pain management by surgeon intervention after surgery; and (5) Patients treated with NSAIDs before surgery.

### Clinical data

The following data were collected: (1) Demographic and clinical baseline data included age, sex, education level, body mass index (BMI), MMSE, American Society of Anesthesiologists (ASA) scores, and medical history; (2) Clinical pathological parameters included surgical type, surgical time, estimated blood loss, visual analog scale (VAS) scores at 1, 2, and 7 days after surgery, EQ-5D scores at 7 and 30 days after surgery, grip strength, treatment requirements in the intensive care unit (ICU), transversus abdominis plane (TAP) block, patient-controlled intravenous analgesia (PCIA: sufentanil 150 ug + dezocine 10 mg + 0.9% normal saline to 150 mL; sufentanil 150 ug + 0.9% saline to 150 mL), and medication (NSAIDs or others); and (3) Laboratory testing included blood cell analysis conducted on the first day before and after surgery. All included data were obtained from medical records. The assessments of MMSE, EQ-5D, VAS scores, and grip strength were conducted as a routine part of the perioperative period.

### Cognitive function assessment

The cognitive function was measured one day before surgery and seven days after surgery. These evaluations were independently conducted by two experienced researchers who were unaware of the protocol. The two researchers received professional training and underwent kappa testing before recruiting patients to ensure the reliability of POCD screening results. Neuropsychological testing included MMSE. As previously studied, the screening for POCD was determined by an MMSE score of less than one standard deviation (SD) [15, 16].

### VAS scores and medication assessment

The VAS scores were recorded and assessed by nurse anesthetists on the 1st, 2nd, and 7th nights after surgery. They also collected other analgesic administration data, including its dose and frequency.

During general anesthesia in elderly patients undergoing radical resection of gastrointestinal tumors, NSAID administration involved parecoxib, which was used once in the perioperative period. The usual dose was 40 mg intravenously, 30 min before the end of surgery. The timing of medication may vary in a few cases due to the different medication habits of the anesthesiologists.

### Ethical statement

This retrospective experiment was approved by the Ethics Committee of Xiangya Third Hospital (No: Fast | 21011). The study was conducted in accordance with the ethical standards of the 1964 Declaration of Helsinki and its later amendments.

### Statistical analysis

Data were analyzed using SPSS software (version 25.0; SPSS Inc., Chicago, IL, USA). The Shapiro–Wilk test was used to determine the distribution of continuous variables, and appropriate tests were employed accordingly. If the variables followed a parametric distribution, classified and continuous data were represented by numbers (percentages, *n*%) and means (SDs). The incidence rates between groups were compared using the chi-square test or Fisher’s exact test. Student *t*-tests were performed to compare continuous data. If the distribution was non-parametric, continuous variables were presented as the median and interquartile range, and non-parametric tests were used to analyze the data. Statistical significance was set at a bilateral *P* value of < 0.05. The average filling method was used for the statistical analysis of missing data.

## Results

### Demographics and clinical characteristics

**Figure 1.**
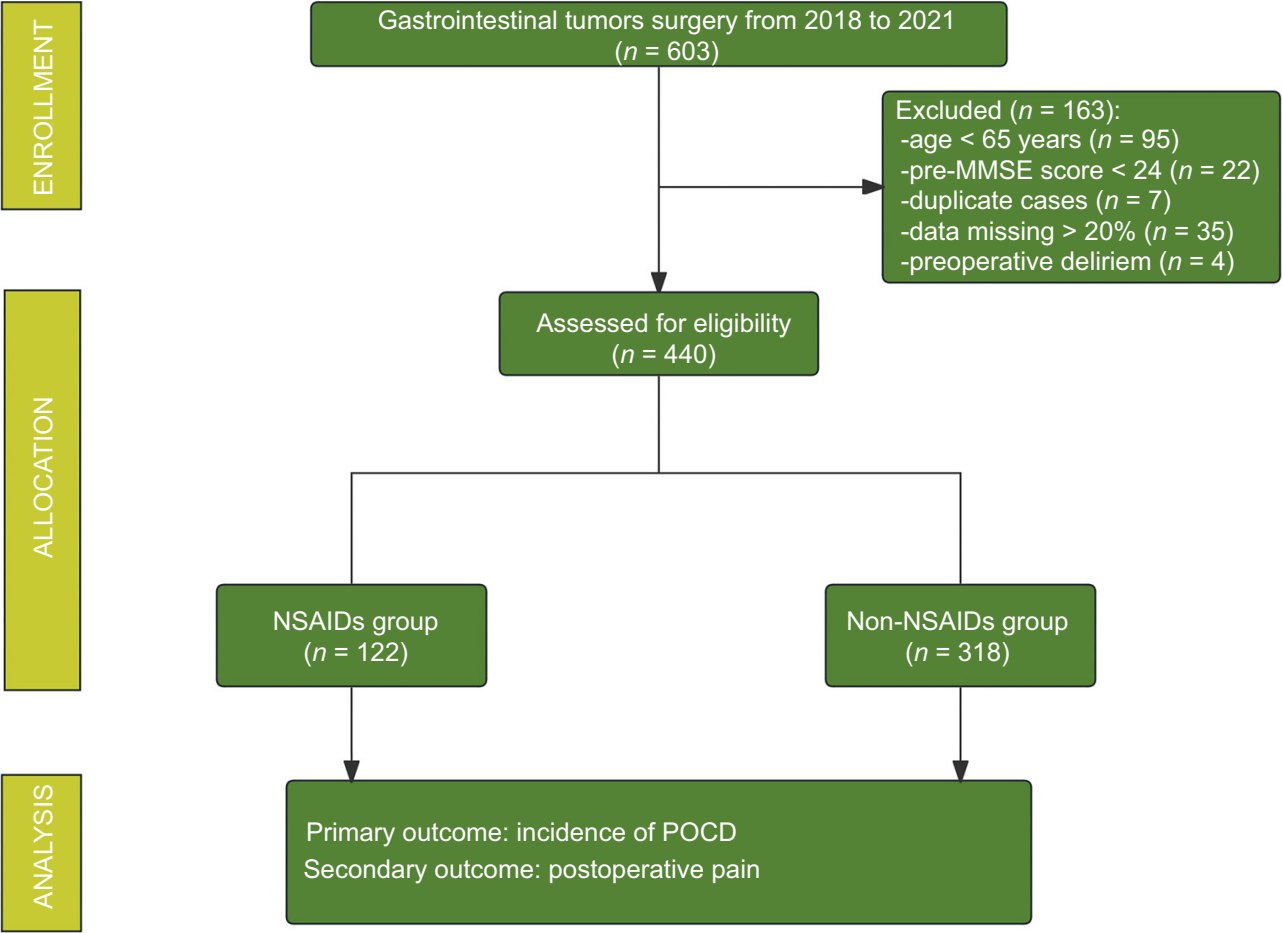
**Flowchat of study selection.** POCD: Postoperative cognitive dysfunction; NSAIDS: Non-steroidal anti-inflammatory drugs; MMSE: Mini-mental state examination.

Among the 440 selected patients, there were 122 patients in the NSAIDs group and 318 patients in the non-NSAIDs group. According to the inclusion and exclusion criteria, the enrollment process of patients is shown in Figure 1. No significant differences were observed in other clinical baseline data (*P* > 0.05) (Table 1).

**Table 1:** Demographic and baseline data associated with postoperative analgesia with NSAIDs in elderly patients with gastrointestinal tumors

**Item**	**NSAIDs group (*n* ═ 122)**	**Non-NSAIDs group (*n* ═ 318)**	***P* value**
Age (y)	70.69 ± 5.05	70.82 ± 4.69	0.858
Male (*n*, %)	90(73.80%)	220(69.20%)	0.504
BMI (kg.m^--2^)	21.94 ± 3.37	22.28 ± 2.86	0.453
ASA (*n*, %)			0.232
II	28(23.00%)	92(28.90%)	
III	94(77.00%)	220(69.20%)	
IV	0(0.00%)	6(1.90%)	
Education (*n*, %)			0.455
Illiteracy	8(6.60%)	10(3.10%)	
Elementary school	94(77.00%)	232(73.00%)	
High school	14(11.50%)	50(15.70%)	
College or higher	6(4.90%)	26(8.20%)	
Preoperative MMSE score	26.79 ± 1.66	26.82 ± 1.81	0.908
Grip strength	26.51 ± 7.06	24.73 ± 6.64	0.083
Medical history (*n*, %)			
Hypertension	50(41.00%)	132(41.50%)	0.943
Diabetes	20(16.40%)	46(14.50%)	0.720
Cerebrovascular disease	8(6.60%)	34(10.70%)	0.350
Cardiovascular disease	22(18.00%)	34(10.70%)	0.144
Chemotherapy	4(3.30%)	10(3.10%)	0.960
Smoking (*n*, %)	40(32.80%)	114(35.80%)	0.670
Alcohol consumption (*n*, %)	24(19.70%)	62(19.50%)	0.977

*P < 0.05. ASA: American Society of Anesthesiologists; BMI: Body mass index; MMSE: Mini-mental state examination; NSAIDs: Non-steroidal anti-inflammatory drugs.

### Effect of NSAIDs on POCD and health status

Our results demonstrated that there was no statistically significant difference in the perioperative MMSE scores between the NSAIDs and non-NSAIDs groups (*P* > 0.05). There were 180 patients with POCD, resulting in an incidence of 40.91%. The incidence of POCD was 42.60% and 40.30% in NSAIDs and non-NSAIDs groups, with no statistically significant difference (*P* ═ 0.749). In addition, no statistically significant differences were observed in the EQ-5D scores and postoperative inflammation levels between the two groups (Table 2).

**Table 2:** Effect of NSAIDs on POCD and health status in elderly patients with gastrointestinal tumors

**Item**	**NSAIDs group** **(*n* ═ 122)**	**Non-NSAIDs group** **(*n* ═ 318)**	***P* value**
*MMSE score*			
Preoperative MMSE score	26.79 ± 1.66	26.82 ± 1.81	0.908
Postoperative MMSE score	25.56 ± 2.54	25.46 ± 2.61	0.801
POCD	52(42.60%)	128(40.30%)	0.749
*EQ-5D score*			
Preoperative EQ-5D score	83.14 ± 9.22	81.25 ± 12.40	0.280
7th day after surgery	69.02 ± 11.50	66.51 ± 15.82	0.197
30th day after surgery	70.44 ± 15.83	71.58 ± 17.54	0.659
*Laboratory test*			
Pre-WBC levels (×10^9^/L)	6.14 ± 1.80	6.22 ± 2.05	0.783
Post-WBC levels (×10^9^/L)	10.73 ± 3.23	11.25 ± 3.95	0.365

*P < 0.05. MMSE: Mini-mental state examination; POCD: Postoperative cognitive dysfunction; EQ-5D: Quality-of-life EuroQol-5 dimensions; WBC levels: White blood cells levels; NSAIDS: Non-steroidal anti-inflammatory drugs.

### Administration of NSAIDs reduced early postoperative acute pain

There were no significant differences in the choice of postoperative analgesia mode between patients in the NSAIDs and non-NSAIDs groups (*P* > 0.05; Table 3). Our results suggested that a single intraoperative administration of NSAIDs could reduce early postoperative acute pain (*P* < 0.001). There were no significant differences in MMSE scores between the two groups at seven days after surgery.

**Table 3:** Effect of NSAIDs on postoperative pain in elderly patients with gastrointestinal tumors

**Item**	**NSAIDs group (*n* ═ 122)**	**Non-NSAIDs group** **(*n* ═ 318)**	***P* value**
Postoperative analgesia (*n*, %)			0.634
PCIA	48(39.30%)	124(39.00%)	
TAP	6(4.90%)	6(1.90%)	
PCIA+TAP	60(49.20%)	172(54.10%)	
None	8(6.60%)	16(5.00%)	
VAS score			
1st day after surgery (activity)	2.55 ± 1.49	4.11 ± 1.54	<0.001*
2nd day after surgery (activity)	2.43 ± 1.27	3.49 ± 1.38	<0.001*
7th day after surgery (activity)	2.83 ± 1.20	3.00 ± 1.56	0.398

*P < 0.05. PCIA: Patient-controlled intravenous analgesia; TAP: Transverse abdominis plane block; VAS: Visual analog scale; NSAIDS: Non-steroidal anti-inflammatory drugs.

### Analgesia with NSAIDs did not increase the rate of negative events

Our study found no statistically significant difference between patients in the NSAIDs and non-NSAIDs groups in terms of postoperative ICU treatment rates, intraoperative blood loss, and 30-day postoperative mortality (Table 4). This indicates that the reasonable administration of perioperative NSAIDs could reduce early postoperative acute pain in patients without significant adverse effects, which was beneficial to patients to a certain extent.

### Effect of perioperative analgesia strategy on postoperative pain and cognitive function

**Figure 2.**
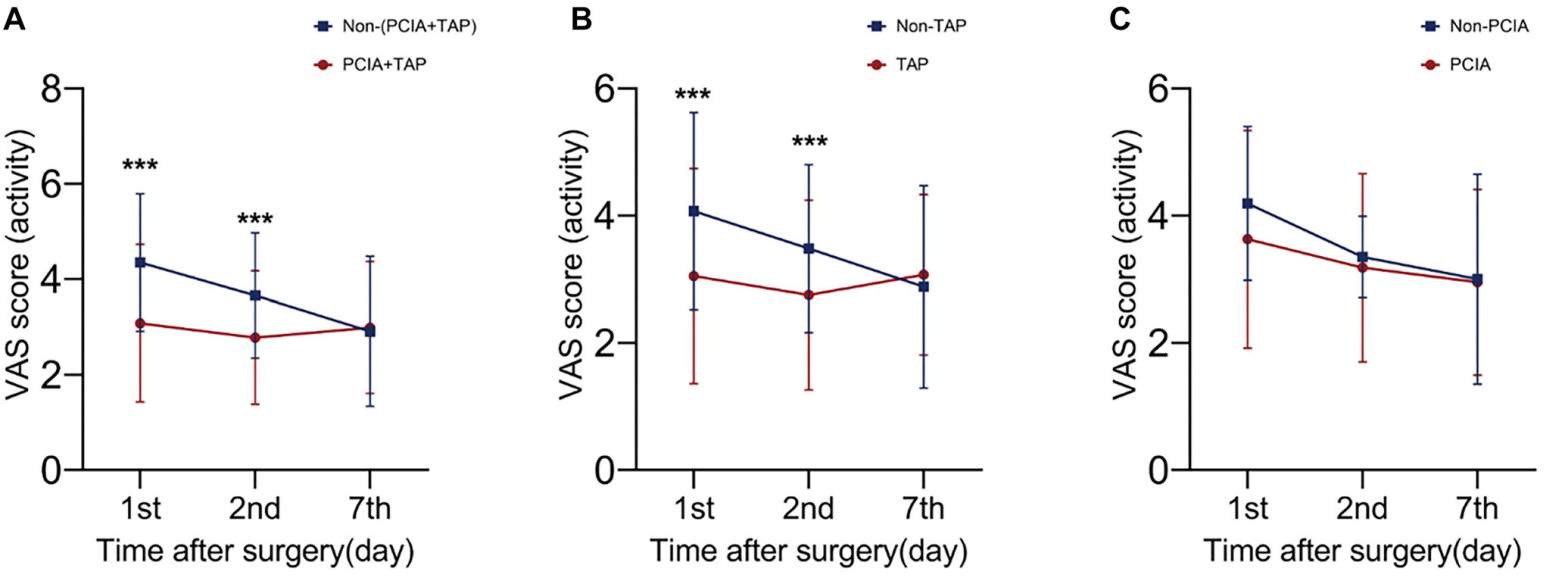
**Effect of perioperative analgesia strategy on postoperative pain.** (PCIA+TAP) vs non-(PCIA+TAP), TAP vs non-TAP, and PCIA vs non-PCIA in VAS scores. PCIA: Patient-controlled intravenous analgesia; TAP: Transverse abdominis plane block; VAS: Visual analog scale.

**Figure 3.**
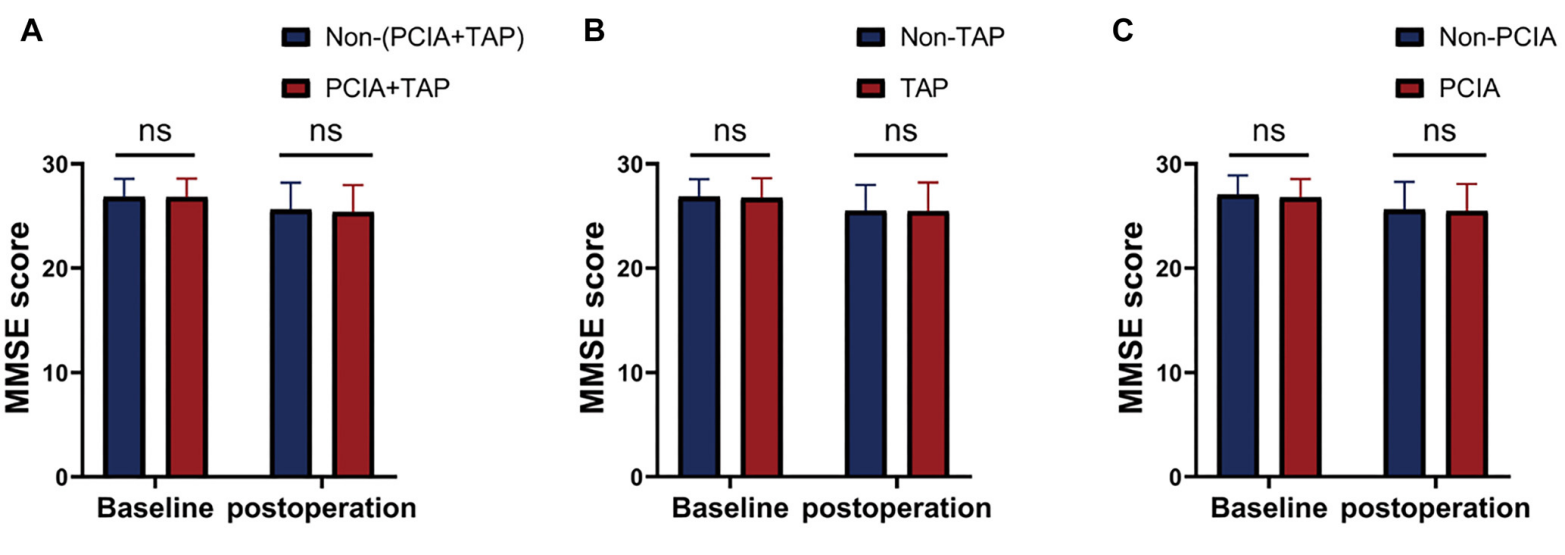
**Effect of perioperative analgesia strategy on cognitive function.** (PCIA+TAP) vs non-(PCIA+TAP), TAP vs non-TAP, and PCIA vs non-PCIA in MMSE scores. ***P < 0.001. PCIA: Patient-controlled intravenous analgesia; TAP: Transverse abdominis plane block; VAS: Visual analog scale.

VAS scores in the (PCIA+TAP) group were significantly lower than those in the non-(PCIA+TAP) group on the first and second day after surgery (*P* < 0.001). Our study also found significant differences in VAS scores between the TAP group and the non-TAP group in the early postoperative period (*P* < 0.001). There were no significant differences in VAS scores between the PCIA group and the non-PCIA group (Figure 2). No statistical differences were observed in postoperative MMSE scores in (PCIA+TAP) vs non-(PCIA+TAP), TAP vs non-TAP, and PCIA vs non-PCIA (*P* > 0.05; Figure 3).

## Discussion

The main finding of the current study was that the administration of NSAIDs was not significantly associated with POCD in these patients. In addition, we found no significant differences in health-related quality of life between the two groups. A single intraoperative administration of NSAIDs could relieve early postoperative acute pain in elderly patients with gastrointestinal tumors without increasing the incidence of adverse events, including intraoperative blood loss, postoperative ICU treatment rates, and mortality within 30 days after surgery.

Regional anesthesia techniques combined with PCIA were effective in pain management. PCIA alone was not effective in postoperative analgesia in patients with gastrointestinal tumors. Multimodal analgesia was the best analgesic strategy for postoperative patients with gastrointestinal tumors.

**Table 4:** Effect of NSAIDs analgesic therapy on postoperative adverse events in elderly patients with gastrointestinal tumors

**Item**	**NSAIDs group (*n* ═ 122)**	**Non-NSAIDs group (*n* ═ 318)**	***P* value**
Blood loss (mL)	193.28 ± 241.27	246.60 ± 336.53	0.260
ICU therapy	8(6.60%)	28(8.80%)	0.785
Mortality (30 days after surgery)	2(1.60%)	8(2.50%)	1.000

*P < 0.05. ICU: Intensive care unit; NSAIDS: Non-steroidal anti-inflammatory drugs.

Our study demonstrated that the administration of NSAIDs was not associated with POCD in elderly patients undergoing radical resection of gastrointestinal tumors, which is contrary to the findings of some previous studies. Kamer et al. [17] reported that meloxicam administered 24 h after splenectomy improves memory function in mice. Additionally, a meta-analysis of eight randomized controlled trials evaluated the effect of parecoxib on the incidence of POCD in elderly patients undergoing orthopedic surgery. In their study, Huang et al. [18] concluded that perioperative treatment with parecoxib could effectively reduce the occurrence of POCD and improve MMSE scores. In these studies, variations in the types of NSAIDs, timing of administration, dosages, and study populations were evident, potentially accounting for the discrepancies in outcomes. The population of our study was elderly patients with gastrointestinal tumors, and based on their fragile gastrointestinal function, our approach of low-dose, single administration may have had less impact on POCD. Notably, several studies have employed different definitions and evaluation methods for POCD. We observed that patients with better neurological conditions were more likely to report pain and consequently received more NSAIDs than those with poorer neurological conditions.

Neuroinflammation is an important underlying mechanism of various neurological diseases [6–8, 12, 13]. Although NSAIDs have shown positive effects on POCD in previous studies, there is currently insufficient evidence from high-quality clinical studies to support definitive conclusions. NSAIDs may offer additional value in multimodal treatment approaches for high-risk patients with POCD; however, high-quality clinical trials are needed to determine whether these effects exist.

NSAIDs are widely used to treat perioperative acute or chronic pain and as preemptive analgesics due to their anti-inflammatory effects and their role in preventing the establishment of injurious pathways, including pericardial and central sensitization [19–21]. Our study found that a single intraoperative application of NSAIDs could alleviate early acute pain following radical resection of gastrointestinal tumors in the elderly, which is consistent with the findings of some prior studies [22, 23]. NSAIDs may be ideal for the treatment of acute pain due to their ability to prevent the establishment of peripheral and central sensitization in nociceptive pathways. Notably, NSAIDs play an important role in the multimodal management of postoperative acute pain. Unlike opioids, NSAIDs present no risk of addiction, and their effects are predictable, resulting in shorter recovery periods, fewer opioid-related adverse reactions, and increased patient satisfaction [24, 25]. However, there are strict restrictions on the indications for NSAIDs administration, which limits their clinical application. COX-2 inhibitors have fewer adverse effects on the digestive tract. The current study concluded that perioperative NSAIDs did not increase the total anastomotic fistula rates in patients; therefore, their safety in patients undergoing radical resection of gastrointestinal tumors has been verified [26, 27]. Nevertheless, adverse reactions associated with NSAIDs use may lead to catastrophic consequences; thus, their indications should be strictly evaluated.

Persistent postoperative pain is a serious problem that affects a patient’s health-related quality of life. However, the exact pathological mechanisms underlying this type of pain remain unknown. Overall, it is considered a complex multifactorial disease that includes aspects of neuroinflammation [28, 29]. Regrettably, at present, there is no clear evidence that perioperative NSAIDs prevent chronic postoperative pain after surgery [30, 31], and our study did not encompass aspects related to postoperative chronic pain. Therefore, exploring the effects of NSAIDs on postoperative chronic pain in elderly patients with gastrointestinal tumors represents a promising avenue for future research.

Some studies have reported the benefits of long-term NSAIDs use, which can reduce the incidence of cancer, tumor metastasis, and recurrence [32–34]. This association is often linked to the dysregulation of COX-2 expression, which is closely associated with carcinogenesis, invasiveness, and angiogenesis. Among prostaglandins, PGE2 stands out as a significant contributor to cancer development, particularly in processes, such as tumor angiogenesis, metastasis, and the inhibition of apoptosis. Theoretically, synthetic inhibition of PGE2 could directly lead to a decline in tumor survival and proliferation [35–37]. However, current evidence for the effect of perioperative short-term NSAIDs therapy on cancer recurrence after major surgery remains inconclusive. Although the immune-based perioperative antitumor effects of NSAIDs have shown promise in observational and retrospective studies, there is no high-quality clinical research evidence to support these effects [38–42]. It is important to note that our study specifically focused on the effect of NSAIDs on 30-day postoperative mortality and did not address adverse outcomes associated with long-term tumor recurrence. Nevertheless, this critical question warrants attention in future research endeavors.

This study has certain limitations. Firstly, residual confounding is a major limitation of this paper, though we used strict inclusion criteria and restrictions to minimize confounding bias. Secondly, our study only covered a subset of the potential adverse events associated with NSAIDs and did not analyze postoperative anastomotic fistulas, asthma, prolonged coagulation function, or renal impairment. Additionally, this study did not evaluate the effects of NSAIDs on the recurrence and survival rates of elderly patients with gastrointestinal tumors. These aspects warrant exploration in future research endeavors. Lastly, while the MMSE is appropriate as an initial screening tool, it is not suitable for definitively diagnosing POCD.

## Conclusion

In summary, our study indicated that parecoxib was beneficial for elderly patients undergoing radical resection of gastrointestinal tumors, alleviating early postoperative acute pain without increasing the incidence of postoperative adverse events. However, it is necessary to accurately determine the indications for parecoxib and exercise caution in its administration. In addition, we found that parecoxib did not increase the incidence of early postoperative cognitive impairment in elderly patients with gastrointestinal tumors. A single low dose of parecoxib is safe and has important implications for early postoperative acute pain management in elderly patients undergoing major surgery.

## Data Availability

The data associated with the paper are not publicly available; however, these are available from the corresponding author upon reasonable request.
